# A Lumped Parameter Model Suggests That Infusion Studies Overestimate the Cerebrospinal Fluid Outflow Resistance in Normal Pressure Hydrocephalus

**DOI:** 10.3390/brainsci14121242

**Published:** 2024-12-11

**Authors:** Grant A. Bateman, Alexander R. Bateman

**Affiliations:** 1 Department of Medical Imaging, John Hunter Hospital, Newcastle, NSW 2310, Australia; 2 Faculty of Health, Newcastle University, Callaghan Campus, Newcastle, NSW 2308, Australia; 3 School of Mechanical Engineering, University of New South Wales, Sydney, NSW 2052, Australia; alexander.bateman@unsw.edu.au

**Keywords:** cerebral blood flow, cerebrospinal fluid formation rate, blood–brain barrier, glymphatic, infusion study, normal pressure hydrocephalus

## Abstract

Background/objectives: Cerebrospinal infusion studies indicate that cerebrospinal fluid outflow resistance (R_out_) is elevated in normal pressure hydrocephalus (NPH). These studies assume that the cerebrospinal formation rate (CSF_fr_) does not vary during the infusion. If the CSF_fr_ were to increase during the infusion then the R_out_ would be overestimated. Previous estimates of the CSF_fr_ in NPH have suggested a low figure. More recent estimates of the CSF_fr_ suggest that it is increased, indicating it probably varies with measurement technique. This would bring the estimation of R_out_ into doubt. A previous paper using a lumped parameter model suggested the CSF_fr_ could vary with the capillary transmural pressure (TMP) in this disease, suggesting a possible solution to this problem. The current study investigates the possibility that the intracranial pressure manipulation occurring during an infusion study may vary the capillary TMP and CSF_fr_. Methods: A lumped parameter model previously developed to describe the hydrodynamics of NPH was modified to investigate the effect of CSF pressure manipulation during infusion studies and to describe how the CSF_fr_ could vary depending on the technique used. Results: The model indicates the capillary transmural pressure is normal in NPH and increases during an infusion study. CSF drainage at the end of an infusion study similarly increases the capillary TMP and, presumably, the CSF_fr_ by increasing the interstitial fluid production. Conclusions: The model suggests that infusion studies and draining CSF increases the CSF_fr_ in NPH compared to earlier techniques. Allowing for an increase in the CSF_fr_ suggests that infusion overestimates the R_out_ by between 23 and 33%. This study indicates that further research may be required into the utility and accuracy of infusion studies and their ability to diagnose NPH.

## 1. Introduction

Patients with normal pressure hydrocephalus (NPH) present with ataxia, incontinence and dementia [1]. The ventricles are dilated but there is a normal cerebrospinal fluid (CSF) pressure [1]. The brain morphology of NPH suggests an abnormality of either CSF formation or absorption. The relationship between the intracranial pressure (ICP) and CSF hydrodynamics is modelled using Davson’s Equation [2],

(1) $$ICP={CSF}_{fr}\times R_{out}+P_{sss}$$

where ICP is the intracranial pressure, CSF_fr_ is the CSF formation rate, R_out_ is the CSF outflow resistance and P_sss_ is the pressure in the superior sagittal sinus. The equation is based around the suggestion that all of the CSF which is formed is absorbed through the arachnoid granulations and into the venous sinuses. Classically, R_out_ is envisaged to be the ease with which CSF can exit the cranial cavity and enter into the venous system [3]. The CSF system in NPH can be tested using an infusion study [4]. The infusion study consists of placing the patient in the lateral decubitus position. A single lumbar puncture needle is placed. The initial reference opening pressure is measured once the steady state is reached. A mock CSF is then infused at a uniform rate of between 1 and 1.5 mL/min and the ICP is measured once the ICP again reaches a steady state [4]. The R_out_ is calculated as the final pressure minus the initial pressure divided by the infusion rate [4]. The method assumes that the only volume change which occurs during the test is due to the infusion [4]. It can be seen that Davson’s Equation predicts a linear relationship between the ICP and the CSF_fr_, and so the infusion of a mock CSF should raise the ICP in a manner that is directly proportional to the R_out_, provided that the CSF_fr_ and P_sss_ are constants and do not change during the test procedure. Initial studies using animal models indicated that the CSF_fr_ did not change following ICP manipulation in the short term, seemingly confirming the first assumption [5]. However, it should be acknowledged that these animals were normal and therefore had no deficiency in regard to their blood–brain barrier (BBB). To our knowledge, the P_sss_ has never been confirmed to be constant in an infusion study and is just assumed to be so.

One of the first methods used to measure the CSF_fr_ in humans was the Masserman technique [6]. Silverberg et al. used a modified Masserman technique where 3 mL of CSF was removed from a ventricular catheter and the time required to return the ICP to the pre-withdrawal level was determined. The CSF_fr_ was calculated as the volume removed divided by the time it took for the pressure to return to the pre-test level. Patients with Parkinson’s disease served as controls and were compared to patients with acute and chronic hydrocephalus. The mean CSF_fr_ for the controls was 0.42 mL/min, the acute hydrocephalus patients were not significantly different at 0.4 mL/min, and the chronic hydrocephalus patients were reduced at 0.25 mL/min or a 40% reduction compared to the controls [5]. These findings indicated to the authors that down regulation of the CSF_fr_ probably occurred in NPH but that it took some time to occur. The normal findings in acute hydrocephalus seemed to indicate that the CSF_fr_ was unaltered by an acutely elevated ICP in humans, similarly to the animal models already discussed. More recently, Qvarlander et al. utilised a technique whereby the CSF pressure was reduced to zero following an infusion study. In those diagnosed with NPH, the CSF_fr_ was 0.46 mL/min compared to 0.40 from historical controls (*p* = 0.005) or a 15% increase [7]. Most recently Tariq et al., using a LiquoGuard7 device to maintain a zero ICP for 30 min, found the CSF formation rate in NPH to be 1.32 mL/min compared to the controls (provided by pituitary adenoma patients), which were 0.48 mL/min or increased by 175% [8]. We can see the three techniques discussed returned similar results for their controls of 0.42, 0.4 and 0.48 mL/min, suggesting that no apparent systematic errors existed between the techniques. The CSF pressure in middle age averages 11.5 mmHg [9] and the pressure gradient from the CSF to the superior sinus lumen is 4 mmHg, ref. [10] giving a normal sinus pressure by subtraction of 7.5 mmHg [11]. Therefore, placing these values into Davson’s Equation indicates the normal R_out_ will be between 8.3 and 10 mmHg/mL/min by using the control cerebrospinal formation rates from these three studies. It has been suggested that the upper limit of normal in regard to R_out_ in a man is 12 mmHg/mL/min [12]. The Dutch normal pressure hydrocephalus study suggested a good outcome from treatment of NPH was noted in individuals with an average R_out_ of 24 mmHg/mL/min [13] or twice the upper limit of normal. However, the divergent CSF_fr_ findings regarding NPH amounts of 0.25, 0.46 and 1.32 mL/min are problematic in terms of the calculation of R_out_ in NPH. We used an average ICP of 13 mmHg in NPH [5], an unchanged P_sss_ of 7.5 mmHg and the three differing CSF_fr_ values to estimate the R_out_ returns values of 22, 12 and 4.2 mmHg/mL/min. That is either high, normal or low.

A recent lumped parameter modelling study of NPH by the authors suggested that the CSF_fr_ in NPH could be altered by a change in the ICP because the BBB was deficient in this disease (unlike the animal models and controls, as discussed above) and the CSF_fr_ would vary with the pressure gradient across the capillary wall or the transmural pressure (TMP) [14]. A potential solution to the variable CSF_fr_ found by the three techniques would be changes in the capillary TMP, altering the CSF_fr_ during each test. Note that an intact BBB, both in the original animal studies comparing ICP and CSF_fr_ and in the human controls from the three discussed studies, would not allow for a change in CSF_fr_ by any alteration in capillary TMP [14]. The possible variation in the CSF_fr_ in NPH could be tested by altering the original lumped parameter model to estimate the capillary TMP for each technique. Therefore, the purpose of this study is to extend the original lumped parameter modelling study in order to incorporate the CSF formation rate and capillary transmural pressure. Thus, we wish to test the hypothesis that the capillary TMP will alter depending on the ICP manipulations that are carried out and can account for the varied CSF_fr_. Ultimately, this will allow us to suggest which figure is correct and indicate which test will over- or underestimate the R_out_.

## 2. Materials and Methods

A full description of the model can be found in the original paper [14]. A brief description is given to outline the methods used.

### 2.1. Equations

The study utilises Ohms law,

(2) ΔP=Q×R

ΔP, pressure drop; Q, blood flow; R resistance. As the resistances are in series, they are directly additive:

(3) $$R_{art}+R_{cap}+R_{ven}+R_{cuf}=R_{tot}$$

R_art_, arterial resistance; R_cap_, capillary resistance; R_ven_, venous resistance; R_cuf_, venous outflow cuff resistance and R_tot_ is the total resistance. Poiseuille’s equation provides the pressure drop across each of these segments:

(4) ΔP=8μLQπr4

ΔP, pressure drop; µ, the viscosity; L, vessel length; Q, blood flow rate; π; a constant and r, radius. Substituting Equation (2) into (4) and eliminating Q from both sides gives an equation for the resistance in each segment:

(5) R=8 μLπr4

The viscosity, length and π are constants, so the resistance depends only on the change in the vessel radius, i.e.,

(6) $$\Delta R=\Delta r^{-4}$$

The vessel volume is given by the equation for a cylinder:

(7) $$V=L\pi r^{2}$$

V, volume; L, vessel length and r the radius. Given L and π are constants, the change in volume is dependent on the change in radius i.e.,

(8) $$\Delta V=\Delta r^{2}$$

Substituting Equation (8) into Equation (6) gives

(9) $$\Delta R=\Delta V^{-2}$$

The transmural pressure across a vessel wall is given by [15]

(10) $$P_{tm}=\frac{4Eh}{{3R}_{o}}(1-\sqrt{\frac{A_{o}}{A}})$$

P_tm_, transmural pressure; E, Young’s modulus; h wall thickness; R_o_, radius in the stress-free state; A_o_, area in the stress-free state and A is the area following the applied transmural pressure. Previously we showed the venous outflow varies with the transmural pressure using the equation [14]:

(11) $${\Delta TMP}_{ven}=-0.033{{\Delta V}_{ven}}^{2}+7.49\times {\Delta V}_{ven}-3.44$$

ΔTMP_ven_, normalised change in venous transmural pressure and ΔV_ven_ is the change in venous volume.

### 2.2. Model Input Parameters

The input parameters are unchanged from the previous study [14]. The brain size is 1500 g. The baseline cerebral blood flow (CBF) is 50 mL/100 g/min [16]. The baseline arterial inflow is 750 mL/min, the arterial inflow pressure is 100 mmHg [17]. The pressure before the capillaries is 32 mmHg [18]. The pressure after the capillaries is 15 mmHg [19]. The normal CSF pressure is 11.5 mmHg [9] with a gradient pressure of 4 mmHg from the CSF to the superior sinus [10]. The sinus pressure is 7.5 mmHg [11]. The transmural pressure in the subarachnoid cortical veins in primates is 2.5 mmHg [20]. The pressure just before the outflow cuff is 14 mmHg.

The total cerebral blood volume (CBV) is 51 mL [14]. The arterial component of the CBV [21] is 12.8 mL. The capillaries occupy 53% of the remainder [22] or 20.3 mL. The venous blood volume is 17.9 mL.

The regression for the outflow resistance is [23]

(12) $$R_{out}=0.075\times Age+9.88$$

Therefore, a normal R_out_ for this study is 13 mmHg/mL/min. The normal CSF formation rate is 0.40 mL/min [24].

### 2.3. Vessel Responses to Transmural Pressure Variations

The arterial resistance depends only on the arterial muscle tone. The arterial transmural pressure has no effect because the arterial pressure is always much higher than the ICP.

In the capillary bed, the vessels are purely passive conduits [25] and react only to their transmural pressure. In a rat model, a reduction in the CBF did not change the capillary size [26]. A maximal increase in the CBF increased the capillary volume by 44% [26]. To simplify the model, the volume of the capillaries was varied between normal and maximally dilated as a linear function of the transmural pressure, i.e., a 1.7% increase in volume occurred for each 1 mmHg pressure rise. Below a TMP of 12 mmHg, the volume is unchanged at 20.3 mL, and above a TMP of 37.9, the elastic limit is reached and the volume is set to 29.2 mL.

The veins also alter their size depending on their transmural pressures. In the previous modelling study [14], the function for the outflow veins was found to be summarised by Equation (11).

The outflow cuff collapse is passively modulated by the transmural pressure between the ICP and the sinus pressure, which is usually negative [27]. The segment is very short and is mostly collapsed with physiological ICPs. Therefore, the change in volume of this segment will be ignored in this model despite the cuff being dilated in the drainage model. Its resistance will be taken into consideration. In the previous study, four cuff transmural pressures resulted in 4 differing resistances [14]. Plotting these points gives a straight line with R^2^ of 0.998. Thus, giving Equation (13):

(13) $$R_{cuf}=-2.71\times {TMP}_{cuf}+0.008$$

R_cuf_, the cuff resistance and TMP_cuf_ is the cuff transmural pressure.

The sagittal sinus pressure will be kept constant in this study.

## 3. Results

### 3.1. Varying Blood Flow in NPH

**Figure 1:**
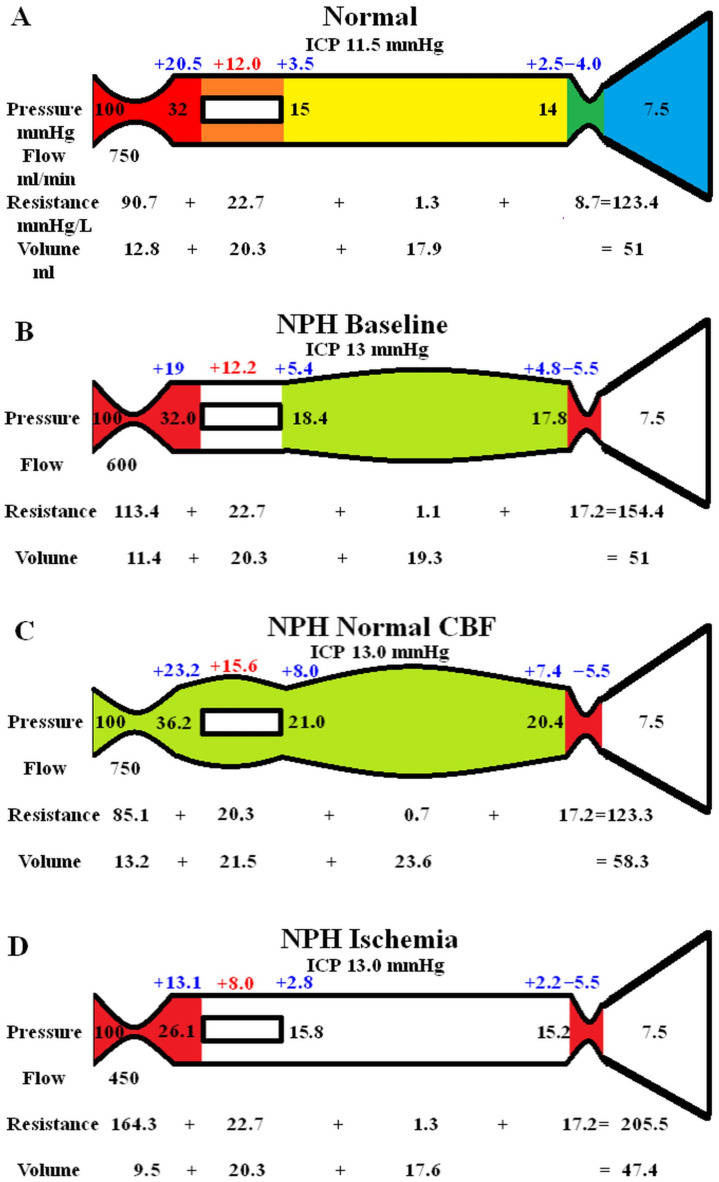
Results of modelling changes to blood flow in NPH. (**A**) Shows the normal findings. Note. ICP: intracranial pressure; mm: millimetres of mercury; mL: millimetres; NPH: normal pressure hydrocephalus. (**B**) Shows the baseline NPH findings; the red area indicates an increase in resistance in the artery and vein cuff and the green indicates decreased resistance in the veins. (**C**) Shows the findings in NPH following an instantaneous increase in blood flow back to normal, with the green area showing a reduction in resistance in the arteries and veins and the red area showing an increase in the outflow cuff resistance. (**D**) Indicates the findings in NPH following a reduction in blood flow of 40% below normal with increased resistance in the arteries. The red areas indicate increased resistance compared to the normal. (**A**,**B**) have been reproduced from [14] under a CC BY 4.0 commons licence.

The original study findings in NPH suggested the brain elected to be ischemic, most likely to limit the capillary TMP and CSF_fr_ [14]. We undertook an initial modelling study to see how an instantaneous change in CBF would alter the capillary TMP to further investigate this effect. These findings are summarised in Figure 1. In Figure 1A, the five segments are the arterial (red), capillary (orange), vein (yellow), outflow cuff (green) and the sinus (blue). The pressures are within the coloured vessel. The arterial inflow volume passes through each segment in sequence. The resistances are shown below the vessels. The cerebral blood volume (CBV) values are indicated below the resistances. The blue numbers are the transmural pressures. The red figure is the average capillary TMP. Figure 1B–D represent the effects of the differing alterations in CBF by varying the inflow resistance. The red segments represent the areas of increased resistance and the green reduced resistance compared to Figure 1A.

In Figure 1B, the baseline findings in NPH found in the original study have been reproduced [14]. The arterial inflow was reduced from normal by 20%, as per the literature. The literature also required the average cerebral blood volume to be unchanged from normal, and the only available result to achieve this was a doubling in the venous cuff resistance and a 25% increase in the artery resistance because these resistances altered the segment volumes to exactly match each other and therefore cancel out. Further information is available in the original study [14].

Figure 1C models an instantaneous return in the CBF to the normal value of 750 mL/min. This required a reduction in the inflow resistance. The resistance across the outflow cuff is unaltered because it only depends on the pressure gradient across its wall, which is unaltered at −5.5 mmHg. The effect of the increase blood flow is to increase the pressure in the veins upstream from the outflow cuff; therefore, the veins dilate compared to the baseline using Equation (11). The vein dilatation reduces the vein resistance using Equation (9). The combination of the increased venous pressure together with the reduced arterial resistance increased the pressure on either side of the capillaries; therefore, the average capillary TMP increased by 28% compared to the NPH baseline. The capillary resistance and volume were adjusted to suit. Note, this is an instantaneous finding, as the model is not expected to be in a steady state. The increased-capillary TMP would be expected to increase the CSF_fr_ and lead to an increased ICP, which would further compress the outflow cuff, increasing its resistance, i.e., a positive feedback loop. A stable system would require a new ICP to be found, which could possibly only occur once all of the arterial dilatation was exhausted.

Figure 1D models the effect of instantaneously reducing the CBF by 40% below normal to 450 mL/min by increasing the inflow resistance by 45%. Again, the outflow cuff resistance is unchanged because the pressure across its wall is unchanged. The pressure across the outflow cuff drops because the blood flow is less. The vein TMP drops and the vein reduces in volume back toward the normal value. The increased arterial resistance increases the pressure drop across this segment and the pressure on either side of the capillaries reduces the average capillary TMP by 34%. Note that, at steady state, the ICP would also be affected by this change as the reduced-capillary TMP would be expected to reduce the CSF_fr_ and, therefore, the ICP further dilating the outflow cuff, further reducing the ICP. The effect of a profound reduction in CBF was modelled but has not been depicted in Figure 1. A 57% reduction in CBF reduced the venous TMP to 0.1 mmHg (the lowest level before a significant vein collapse would occur); additionally, the capillary TMP was reduced to 4.4 mmHg and the vein volume was reduced by 12.8% below the normal value.

### 3.2. Varying the ICP in NPH

**Figure 2:**
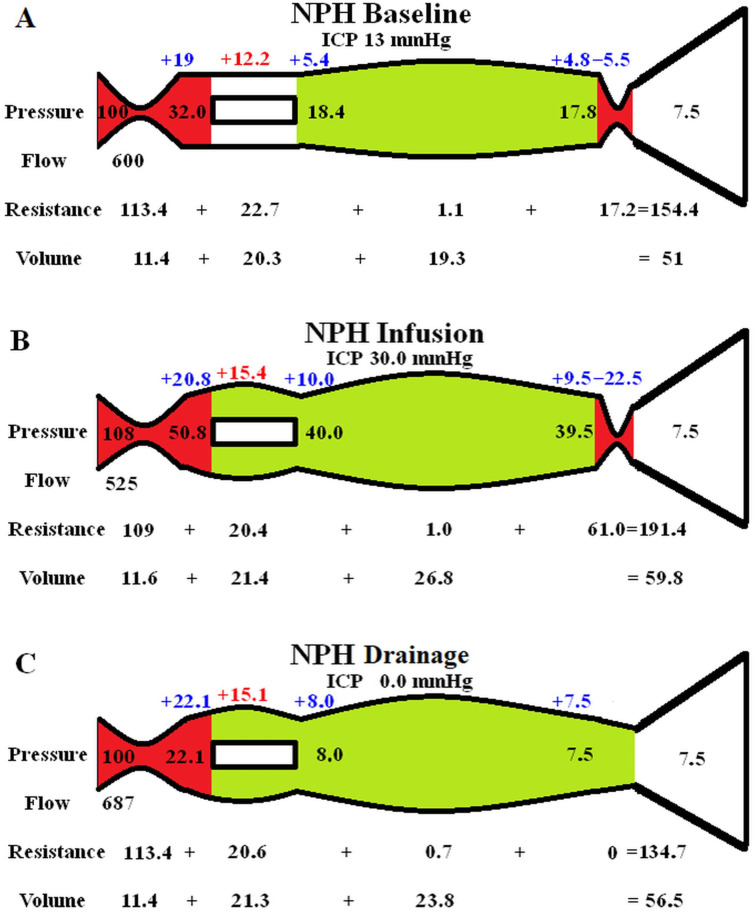
Modelling of changes to ICP in NPH. (**A**) shows the baseline NPH findings, as seen above in Figure 1B. Red depicts increased resistance and green lower resistance compared to normal. (**B**) shows the findings in NPH following an infusion study, with the green area highlighting a reduction in resistance in the capillaries and veins and the red area showing an increase in the arteries and outflow cuff compared to normal. (**C**) shows the findings in NPH following CSF drainage to lower the ICP to zero. The outflow cuff resistance is abolished, reducing the venous and capillary pressure, but the ICP has a greater reduction, leading to an increase in capillary TMP. Red depicts increased resistance and green lower resistance compared to normal. Note, TMP: transmural pressure. (**A**) has been reproduced from [14] under a CC BY 4.0 commons licence.

The effect of varying the ICP in NPH had been modelled in Figure 2. Again, Figure 2A shows the baseline NPH findings from the original study [14]. Figure 2B models an infusion study. Momjian et al. studied 12 patients with NPH; the baseline ICP was 11.1 mmHg and the ICP increased by 17.3 mmHg to 28.4 mmHg during the study [28]. During the infusion, the arterial blood pressure increased by 8 mmHg. The baseline CBF was 14% below the controls and the CBF reduced by a further 10% following infusion. The P_sss_ was not measured. The calculated R_out_ was 17.6 mmHg/mL/min [28]. Using Davson’s Equation, we can calculate the average infusion rate to be approximately 1 mL/min. Thus, to match these findings in the model, the arterial inflow pressure was increased by 8 mmHg, the ICP was increased by 17 mmHg and the arterial inflow was reduced to 525 mL/min. The effect of these changes was to significantly increase the outflow cuff resistance because the pressure gradient across its wall increased by four times compared to the baseline. This significantly increased the vein pressure and dilated the veins further. The arterial inflow resistance needed to be minimally reduced (−3.9%) to maintain the flow at the pre-set level, but this was not considered to be a significant change. The overall effect was an increase in capillary TMP of 26% above baseline.

Figure 2C is the effect of reducing the ICP to zero by drainage. In a review of the change in CBF following tap tests, Owler and Pickard suggested that there was an equal likelihood of a decrease in the CBF as well as an increase [29]. However, in a study comparing 13 patients with probable NPH who had at least one positive tap test or infusion study result vs. 10 patients who did not, the deep white mater CBF increased by 46% following the tap test in the positive patients whilst the negative patients reduced their CBF by 21% [30]. Most tap tests will not reduce the pressure to zero, perhaps underestimating the improvement in the CBF. A reduction in ICP to zero has a significant effect on the outflow cuff resistance, which will effectively drop it to zero due to maximal dilatation. In order for no change in the global CBF to occur, the arterial resistance would need to significantly increase by approximately 15% to balance the reduced outflow resistance. This seems unlikely in NPH but possible in the negative tap test patients (probably Alzheimer’s disease) where the CBF was reduced. It was decided not to alter the arterial resistance from baseline and see how the blood flow responded. The effect was for the blood flow to increase by 14.5% above baseline, which seemed reasonable. Although the pressures before and after the capillaries dropped, they did not drop by as much as the ICP. Therefore, the capillary TMP was elevated and was not significantly different to the infusion model, with an increase of 24% above baseline.

A third model was performed to gauge the middle ground between normal and the infusion study ICP. The ICP was set to 21.5 mmHg, the arterial pressure was increased to 104 mmHg and the blood flow was set to 563 mL/min. The results are not depicted in Figure 2 but the capillary TMP was 13 mmHg.

## 4. Discussion

### 4.1. Variation in R_out_ in NPH

As discussed in the introduction, there seems to be some variability in either the CSF outflow resistance (R_out_) or the CSF formation rate (CSF_fr_) in NPH depending on the technique used to measure them. This conjecture could be suggested to be theoretical and one could dismiss both of the studies by Silverberg [5] and Tariq [8] as outliers to resolve this dilemma. However, this is not the only time that this problem has arisen. Sundstrom et al. measured the R_out_ in a cohort of 20 patients with NPH using three differing methods on each patient in turn, namely constant pressure infusion, constant flow infusion and bolus infusion [31]. They also used each method on an experimental model with known outflow characteristics. In the experimental model, there was no significant difference between methods overall. In the NPH patients, the constant pressure infusion gave a mean R_out_ of 16.0 mmHg/mL/min (i.e., high), the constant flow averaged 13.0 mmHg/mL/min (just above normal) and the bolus method resulted in 9.8 mmHg/mL/min (normal). The rise in ICP in the constant pressure method was always greater than 18 mmHg, the constant flow method increased the ICP by approximately 18 mmHg and the bolus method was said to result in much less. The bolus method required only 4 mL of fluid to be injected, and the estimate was taken from the midpoint of the relaxation curve [31]. In the case of patients with idiopathic intracranial hypertension, in those with the most incompliant cranial systems, removal of 3 mL of fluid would decrease the ICP by 2.6 mmHg, while removing 4 mL would reduce it by 3.5 mmHg [32]. The cranial system becomes more incompliant as the ICP is increased; however, even if the ICP were to increase by 5 mmHg overall during the bolus method, the measurement would still be taken at about 2.5 mmHg above normal, being much lower than the other two studies. Thus, altering the ICP may be affecting the measurement of the R_out_.

### 4.2. Variation in CSF_fr_ with Capillary TMP

In our previous study, we suggested that the CSF_fr_ could vary with the capillary TMP if the BBB was open [14]. It has been found that the CSF_fr_ does not change with the ICP and remains a constant [33]. The CSF is produced from differing regions within the brain. Seventy percent comes from the choroid plexus, 18% comes from the capillaries and 12% comes from the glucose metabolism [34]. The only possible instantaneously variable component of the CSF production would come from the capillary bed, but this is excluded if the BBB is intact. A long-term reduction in the flow rate would require some down regulation of the choroid plexus production. Capillary CSF production or absorption is expected to follow the Starling forces relationship. This is modelled using the following equation:

(14) $$J_{cap}=L_{cap}[\left\{P_{cap}-P_{CSF}\right\}-\sigma_{cap}\left\{\pi_{cap}-\pi_{CSF}\right\}]$$

where J_cap_ is capillary fluid flow rate; L_cap_ is capillary hydraulic conductivity; P_cap_ − P_csf_ is capillary TMP; σ_cap_ is the osmotic reflection coefficient and π_cap_ − π_csf_ is the osmotic pressure gradient across the capillary. This incorporates both the salt (colloid) osmotic and the protein (oncotic) pressures [35]. Note that, at steady state, there is no measurable difference in hydrostatic pressure between the CSF and the brain parenchyma [36,37]. When the BBB is intact, the cerebral capillaries have hydraulic conductivities of 2–3 orders of magnitude less than systemic capillaries [38] and the osmotic reflection coefficient is 1 [35]. In the brain, the colloid (salt) osmotic pressure of the plasma and interstitial spaces are identical at approximately 5100 mmHg [35]. The protein oncotic pressure within the plasma is 25 mmHg, and this is normally negligible in the brain interstitial space due to the low protein levels [35]. This suggests that the total resultant pressure between the capillary bed and CSF would be −13 mmHg and, therefore, tends to absorb free water back into the capillaries if the hydraulic conductivity is not so low. As a secondary effect, if there were increased capillary water flow from an increase in capillary TMP there would rapidly arise an increase in the osmotic pressure difference between the plasma and the brain interstitium. This occurs as the salt and proteins do not follow the water due to their high osmotic reflection coefficient. Thus, there would be an increase in osmotic pressure in the capillary plasma and a decrease in the interstitium, further opposing the hydrostatic pressure difference [35]. This feedback control fails when the BBB breaks down. The net result is an increase in brain water and the ICP [35]. This happens because the hydraulic conductivity is increased and the osmotic reflection coefficient for salt approaches zero. The protein osmotic reflection coefficient of the brain can approach that for the peripheral tissues, i.e., 0.93, meaning up to 7% of the protein may be filtered from the capillaries [35]. In NPH, there is a significant disruption of the BBB with protein leakage. In hydrocephalus, there is capillary blood–brain barrier dysfunction, with increased vesicular and vacuolar transport, open inter-endothelial junctions, thin and fragmented basement membranes, and discontinuous perivascular astrocytic end-feet [39]. Eide and Hansson found, in pathological specimens of the cortex taken from NPH patients, that there was preferential extravasation of fibrinogen in the cortex from capillaries with damaged and open BBBs [40]. The effect of protein leakage into the interstitium is to lessen the effect of the oncotic pressure difference between the plasma and interstitial space. Thus, at some point, raising the capillary TMP will promote an increase in the CSF_fr_, and a further increase in capillary TMP would produce a further increase in the CSF_fr_. Similarly, decreasing the capillary TMP may promote CSF reabsorption with an open BBB.

In a previous paper, we suggested that reducing the average capillary TMP in NPH by allowing a 20% overall decrease in cerebral blood flow (CBF) may be a harm minimisation strategy in that the TMP and CSF_fr_ would be reduced [14]. Figure 1 explores this suggestion further. The baseline capillary TMP in hydrocephalus is not significantly different to normal at 12.2 mmHg (Figure 1A,B). Increasing the CBF back to normal (Figure 1C) increased the capillary TMP to 15.6 mmHg and significantly increased the ICP via a positive feedback loop. Decreasing the CBF further to 40% below normal decreased the capillary TMP to 8 mmHg and significantly reduced the CSF_fr_ and, therefore, the ICP. The average reduction in CBF of 20% thus seems to be a compromise between the resultant ICP and maintaining overall brain health by minimising the ischemic damage.

### 4.3. Differences Between Cortex and Periventricular White Matter in NPH

**Figure 3:**
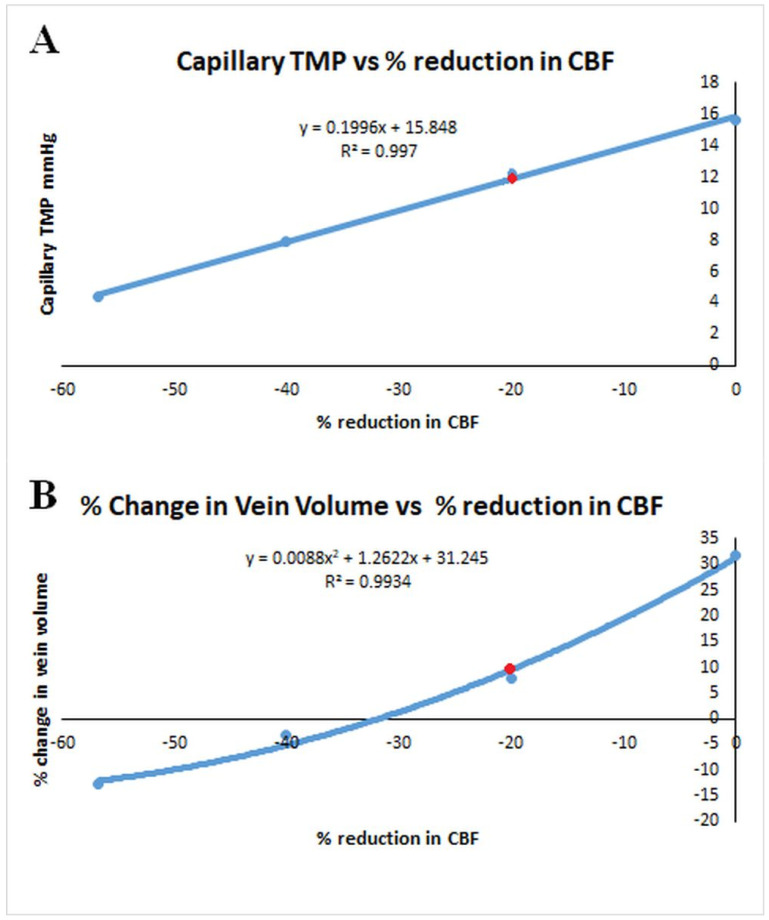
Vascular changes in NPH compared to the cerebral blood flow. (**A**) A graph of the four calculated capillary transmural pressures vs. the cerebral blood flow for the four studies obtained from the initial modelling. The red dot is the baseline finding in NPH. Note, increasing the CBF above baseline increased the capillary TMP, while decreasing the CBF did the opposite in a linear fashion. (**B**) A graph of the four calculated venous volumes vs. the cerebral blood flow for the four studies obtained from the initial modelling. The red dot is the baseline finding in NPH. Note, increasing the CBF above baseline increased the venous volume and decreasing the CBF did the opposite in a quadratic fashion.

As discussed, the literature suggests an overall reduction in CBF of 20% at baseline in NPH. However, this reduction is not evenly spread across the entire brain. In a study of naturally occurring hydrocephalus in Cavalier King Charles spaniels, there was a 58% reduction in periventricular white matter CBF with no significant difference in the cortex [41]. In humans, the white matter CBF reduction is not as dramatic. One study indicated that the maximal reduction in CBF occurred just below the ependymal at −67% and returned to normal in the superficial white matter in a logarithmic fashion [28], giving an average reduction of approximately 40%. No variation in CBF was seen across the white matter in the controls [28]. If the global average CBF reduction is 20% but the white matter reduction averages 40%, then we can calculate the average grey matter CBF. Given that the grey matter makes up 65% of the brain, the white matter makes up 35% [42] and the average CBF ratio is 1.7:1 [43], respectively, we therefore can show that the CBF reduction for the cortex must be approximately 13.7%. This suggestion is corroborated by SPECT perfusion imaging in NPH patients, which shows an apparent hyper-perfusion of the high convexity cortex; however, this was later found to represent an increase in grey matter density from compression rather than an increased CBF [44]. The actual CBF in the cortex was reduced by between 7.8 and 25.7% compared to the controls [44]. Our estimate is at the centre of this range. The effect of this will be discussed with the help of Figure 3. Figure 3A is the graphical representation of how the reduction in CBF from the three NPH studies in Figure 1 (together with the non-depicted profound ischemia study) will alter the capillary TMP. As can be seen, the average CBF reduction of 13.7 in the cortex leads to a capillary TMP of 13.1 mmHg, which is 9% above normal and would tend to promote an increase in the production of interstitial fluid. The average reduction in CBF of −40% in the white matter would lead to a capillary TMP of 8 mmHg or a 33% reduction and may promote a net absorption of interstitial fluid and CSF. The increased inflow of fluid at the cortex would be balanced by the outflow at the white matter, so the system would be in a steady state. An increase in interstitial fluid from the cortex is suggested in the literature. Measurement of the CSF flow within the aqueduct in NPH patients indicates a 3–4 times larger CSF volumetric flow rate passing into the ventricles from the subarachnoid space as compared to controls where the flow is in the opposite direction (out of the ventricles) [45]. With regard to the white matter, the large amount of fluid entering the ventricles from the subarachnoid space would increase the amount of parenchymal free water. An MRI study of patients with NPH had a higher free-water fraction in their periventricular hyperintensities compared to control patients despite the controls having similar small-vessel hyperintensities [46]. One may ask, does the free water transit the brain to leave by the cortex? In a rat hydrocephalus model, injected extracellular tracer movement in the cortex was inhibited and the fluid preferentially accumulated in the deep white matter. In control animals, the tracer left the cortex without accumulating in the white matter. The authors concluded that white matter accumulation of the tracer reflected bulk fluid flow to this site from the cortex [47]. Obviously, if the water is travelling both (1) from the cortex to the white matter and (2) from the subarachnoid space to the white matter, then the white matter must be absorbing water into its capillaries as it would have nowhere else to go.

The previously described rat study brings up an interesting finding. Most of a tracer injected into the cortex would be expected to travel into the subarachnoid space over the vertex via the perivenous channels as part of the glymphatic system [48]. The rat study discussed above indicates that the glymphatic system within the cortex is deficient but is probably relatively preserved in the white matter. In humans using intrathecal gadolinium as a tracer, NPH was found to be associated with the ventricular reflux of the tracer and an overall reduced clearance rate of the tracer. The reduced clearance rate indicated to the authors glymphatic dysfunction [49]. Previously, we have suggested that venous dilatation within the venous perivascular space will increase the glymphatic outflow resistance and therefore reduce glymphatic flow [50,51,52]. Figure 3B is a plot of the change in venous volume vs. the change in CBF for the four models studied as per Figure 3A. In NPH, the cortex would have an increase in venous volume of 15.6% and the average white matter vein volume would be reduced by 3.3% compared to normal, suggesting a reduced glymphatic flow superficially and a preserved flow on a deeper level. Inserting a shunt tube has been previously shown to reduce the vein volume by 12.8% globally [14] and, therefore, would be expected to improve glymphatic flow.

### 4.4. Which Test for R_out_ in NPH Is Most Accurate?

**Figure 4:**
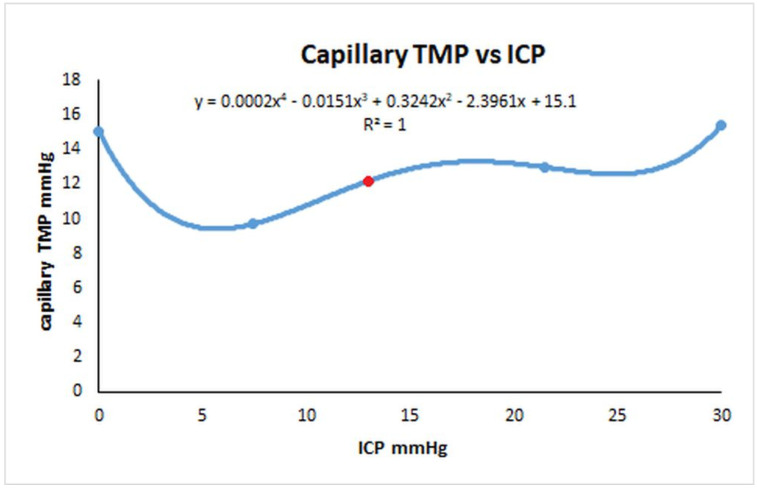
Relationship between the capillary transmural pressure and ICP in NPH. A graph of the change capillary transmural pressure vs. ICP from the data obtained from the second modelling study. The red dot indicates the NPH baseline. The graph is a complex polynomial function but the capillary TMP increases to an identical value at either extreme of the ICP change. The capillary TMP will remain close to the baseline if the ICP is not changed by much either way.

We started this study to try to find which test for R_out_ would be the most accurate and if infusion studies would overestimate it. As discussed in the introduction, infusion studies are hypothesised to measure the outflow resistance across the arachnoid granulations, but what does this mean if the majority of the CSF absorption switches from arachnoid granulations and the deficient glymphatic system to the capillary bed? Is an infusion study attempting to measure the capillary CSF outflow resistance instead? If so, then Davson’s Equation in regard to this disease is not valid. Bonney suggested that, in chronic states of hydrocephalus, any imbalance between CSF production and absorption is transient, with a new equilibrium being reached by either increased CSF absorption through means other than the arachnoid granulations or decreased CSF production [53]. It may be that both an increased absorption through the white matter and a reduction in CSF_fr_ by down regulating the choroid plexus CSF production may occur. Ideally the R_out_ would be measured without varying the ICP at all; however, this is not possible. The response of the capillary TMP to the ICP in NPH is a complex function and has been depicted in Figure 4. In this figure, the capillary TMP vs. the ICP has been plotted from the baseline NPH study, the infusion and drainage studies together with an infusion study model where the ICP was midway between the baseline NPH and infusion models (the findings were described in the results but not depicted). An additional point is provided by the post-shunt model from the previous NPH study [14], which gives the point close to the nadir of the graph. The red dot represents the baseline model. Note, complete drainage to zero ICP and the full infusion study both increase the capillary TMP and probably the CSF_fr_ almost identically. Qvarlander et al. suggest that the CSF_fr_ for drainage (zero ICP) was 0.46 mL/min [7], and the CSF_fr_ for their infusion study (ICP of 30 mmHg) is therefore also likely to be the same. The study estimating the CSF_fr_ by Silverberg et al. only removed 3 mL of fluid [5], and we have previously suggested that the study by Griffith [32] indicates that this would only reduce the ICP by perhaps 3 mmHg; however, the average for the test would only be 1.5 mmHg because the CSF pressure is allowed to rise back to normal during measurement. Silverberg et al. suggested a CSF_fr_ of 0.25 mL/min, and this is likely more accurate than the suggestions of Qvarlander et al. If, during the infusion study by Momjian et al., the CSf_fr_ increased from 0.25 to 0.46 mL/min, then the increase (0.21 mL/min) would need to be added to the infusion rate of 1 mL/min. When we do this, the R_out_ is reduced from 17.6 to 14.3 mmHg/mL/min, indicating it may be overestimated by 23%. In the study by Sundstrom et al. where NPH patients underwent three differing methods to measure R_out_ [31], the bolus method (as previously discussed) would be expected to only increase the ICP by a small amount and, therefore, be the most accurate test. The R_out_ for the infusion study averaged 13 mmHg/mL/min while the bolus method averaged 9.8 mmHg/mL/min, suggesting the infusion method overestimated the R_out_ by 33%.

Finally, we would need to explain the findings by Tariq et al., i.e., that the CSF_fr_ is greatly increased at 1.32 mL/min [8], by using a technique almost exactly the same as Qvarlander et al., who found the same metric to be 0.46 mL/min [7]. The difference is that Qvarlander et al. reduced the ICP to zero immediately after an infusion study where the ICP was raised to about 30 mmHg while Tariq et al. did not. In a preliminary study recently reported as an abstract at the Hydrocephalus 2024 conference, Tariq et al. repeated their LiquoGuard7 study both before, immediately after and then 1–2 days after a standard infusion study. The CSF_fr_ was 1.27–01.67 mL/min before the infusion test, 0.4–0.6 mL/min immediately after the infusion (similar to Qvarlander et al.) and returned to 1.27–1.67 mL/min at 1–2 days after the test [54]. Therefore, something about the infusion study is decreasing the CSF_fr_ compared to the other time points. One possible suggestion is that the infusion study dilates the veins by 32% because of the high ICP (see Figure 3B). This would significantly block the glymphatic system even more than what occurs at baseline in NPH. This glymphatic blockage may take some time to resolve and the CSF_fr_ and glymphatic flow would therefore increase over time. However, Figure 2B,C suggests that both drainage at zero ICP and infusion produces a similar increase in venous diameter, so this is unlikely to be the cause of the difference. Alternatively, it is known that aquaporin 1 (found in the choroid plexus) is down regulated within the choroid in animal models of hydrocephalus [55]. Increased down regulation could occur as a response to an elevation in ICP from the infusion test to limit interstitial fluid production. Human aquaporin 1 has also been shown to rapidly alter its conductance by a reversible membrane tension mediated mechanism [56]. Down regulation or tension-mediated closing of these channels could take some time to reverse and would increase the CSF_fr_ over time.

This study provides new and novel insights into the physiology underlying NPH. We believe it is possible that the hypothesis that the brain is able to vary its perfusion pressure to alter the effect of a disruption of the blood–brain barrier may have applications in regard to other cerebral pathologies. Infusion studies are currently a well established test to diagnose and to stratify NPH patients for treatment. If these test are flawed, as we suggest, then more research may be required to validate their findings. Ultimately, we hope that our insights will lead to more targeted therapies for this disease-treatment process.

### 4.5. Limitations

Lumped parameter modelling makes many assumptions. Poiseuille’s equation models flow through thin, rigid, circular tubes of a Newtonian fluid without turbulence. We cannot prove that these assumptions hold but, if true, the findings would be accurate. Despite this limitation, this equation is commonly used to model the vasculature within the literature.

In our modelling, we did not vary the sinus pressure as no data were available to allow for this. However, this pressure is always assumed to be a constant in hydrocephalus. Theoretically, the reduction in CBF caused by hydrocephalus could reduce the venous sinus pressure. A sensitivity analysis was performed with a reduction in the sinus pressure of 10%. In the baseline NPH model, this increased the outflow cuff resistance and decreased the arterial and venous resistance, giving an increase in capillary transmural pressure of 7%. In the NPH infusion model, there was very little difference in the capillary transmural pressure because the change in cuff resistance was small due to the large gradient pressure across the cuff to begin with. These findings would tend to suggest the conclusions are unlikely to be significantly altered. Altering the arterial inflow pressure did not change the results because, in order to maintain the same blood flow, the overall and arterial resistance were increased by the same amount. The venous resistances were unchanged as they were set by the transmural pressure across the cuff, which was unaltered. Interestingly, Chabros et al. acknowledge that their reference pressure is usually hypothesised to be the sagittal sinus pressure [3]. The authors then proceed to claim that this correspondence has been refuted in NPH but only referenced their own unpublished results to support this claim [3]. This is presumably because they have measured the SSS pressure to be higher than that required for their model to work. They quote a retrospective study of 858 patients who underwent infusion to diagnose NPH and found the median R_out_ was 12.36 mmHg/mL/min, the median CSF_fr_ was 0.48 mL/min, and the median reference pressure was 4.91 mmHg [3], giving a median ICP of 10.8 mmHg using Davson’s Equation. We are unsure as to what this very low reference pressure corresponds with, as it is 35% less than the sinus pressure (which does correspond to the reference pressure for controls) and the capillary bed pressure is even higher. As discussed, Davson’s Equation is probably invalid in NPH, so the reference pressure is not useful.

Some of the data we required for this model are not available from human studies. In their absence, animal studies were utilised. This is exemplified by the data linking the dilatation of the capillaries to the changes in TMP, which was taken from rodent studies and the normal venous TMP, which was obtained from primate studies. We have no way of knowing if the animal data closely approximate human findings, so this is a limitation.

## 5. Conclusions

In NPH, the brain redirects its CSF absorption from the arachnoid granulations to the deep white matter by a combination of an opening of the blood–brain barrier and a reduction in the capillary transmural pressure. Therefore, the presumed increase in R_out_ at the arachnoid granulations and the brain lymphatics in NPH is balanced by the reduction in outflow resistance across the capillaries, so the overall R_out_ is probably normal. Infusion studies increase the CSF_fr_ during the test, and, if this is not taken into account, the R_out_ will be overestimated.

## Data Availability

The original contributions presented in the study are included in the article, further inquiries can be directed to the corresponding author.
